# Rituximab at lower dose for neuromyelitis optica spectrum disorder: a multicenter, open-label, self-controlled, prospective follow-up study

**DOI:** 10.3389/fimmu.2023.1148632

**Published:** 2023-08-08

**Authors:** Daidi Zhao, Kaixi Ren, Jiarui Lu, Zhiqin Liu, Zunbo Li, Jun Wu, Zhihao Xu, Songdi Wu, Tao Lei, Chao Ma, Sijia Zhao, Miao Bai, Hongzeng Li, Jun Guo

**Affiliations:** ^1^ Department of Neurology, Tangdu Hospital, Air Force Medical University, Xi’an, China; ^2^ Department of Neurology, Xi’an Central Hospital, Xi’an, China; ^3^ Department of Neurology, Xi’an Gaoxin Hospital, Xi’an, China; ^4^ Department of Neurology, Xianyang Central Hospital, Xianyang, China; ^5^ Department of Neurology, Baoji Central Hospital, Baoji, China; ^6^ Department of Neurology, The First Hospital of Xi’an, Xi’an, China; ^7^ Department of Neuroophthalmology, Xi’an Fourth Hospital, Xi’an, China; ^8^ Department of Cardiology, Tangdu Hospital, Air Force Medical University, Xi’an, China

**Keywords:** neuromyelitis optica spectrum disorder, rituximab, low-dose, efficacy, safety

## Abstract

**Objective:**

To address a novel lower-dose rituximab (RTX) therapy strategy based on our clinical experience and assess its efficacy and safety in neuromyelitis optica spectrum disorder (NMOSD).

**Methods:**

A multicenter, open-label, self-controlled, prospective follow-up study. Totally, 108 NMOSD patients were enrolled and a lower-dose RTX strategy was applied including 100 mg weekly for 3 weeks and then reinfusions every 6 months. Annualized relapse rate (ARR), the expanded disability status scale (EDSS) score and length of spinal cord lesions were included to evaluate the efficacy. Side effects were recorded to assess the safety profile.

**Results:**

Of 108 patients, 80 (74.1%) initiated low-dose RTX therapy immediately after acute attack treatment and 33 (30.6%) initiated it after the first attack. During a median treatment period of 35.5 (22.0–48.8) months, significant decreases were observed in median ARR (1.1 [0.8–2.0] versus 0 [0–0.2], *p* < 0.001), EDSS score (3.5 [2.5–4.0] versus 2.0 [1.0–3.0], *p* < 0.001) and spinal cord lesion segments (5.0 [4.0–8.0] versus 3.0 [1.0–6.0], *p* < 0.001). The cumulative risk of relapses significantly decreased during the post- versus pre-RTX period (HR 0.238, 95%CI 0.160–0.356, *p* < 0.001) and on early therapy initiated within 24 months after disease onset versus delayed therapy (HR 0.506, 95%CI 0.258–0.994, *p* = 0.041). No serious side effects were recorded and all the subjects did not discontinue treatment due to RTX-related side effects.

**Conclusion:**

Our research provided evidence supporting the lower-dose RTX strategy in treating NMOSD and reopened the issues of optimal dosage and therapy initiation timing.

## Introduction

Neuromyelitis optica spectrum disorder (NMOSD) is a chronic autoimmune inflammatory disease of the central nervous system mainly characterized by optic neuritis and longitudinal extensive transverse myelitis. Additionally, it may present with syndromes of area postrema, brainstem, diencephalon, and cerebrum (1). Since the discovery of pathogenic antibodies against water channel protein aquaporin-4 (AQP4) (2), it is considered a separate disease entity distinct from multiple sclerosis and has a higher prevalence in East Asian and African populations than in Caucasian population (3, 4). NMOSD is a rare but clinically aggressive disease, where cumulative damages from frequent clinical relapses would result in permanent disabilities and even mortality. Therefore, seeking effective and safe immunosuppressive or immunomodulatory drugs would be necessary to prevent relapses and reduce disability.

Nowadays, only three recombinant monoclonal antibodies have been approved to treat AQP4-IgG seropositive NMOSD given a good efficacy and safety profile confirmed in phase 3 trials, including IL-6R-targeting satralizumab (5, 6), CD19-targeting inebilizumab (7) and complement 5-targeting eculizumab (8). However, the economic costs and treatment compliance need to be taken into consideration when a long-term therapy would be applied for NMOSD. Moreover, no access or difficult access to these drugs in China limits their wide use and also promotes the attempts for alternative treatments. In the past two decades, off-label use of conventional immunosuppressive drugs such as mycophenolate mofetil (MMF) or azathioprine (AZA) has been extensively accepted (9, 10), but long-term daily medication affects treatment compliance to some extent. Instead, some biologic agents such as rituximab (RTX) have been increasingly applied to treat NMOSD (11–13) and are more effective than AZA or MMF in the prevention of subsequent clinical relapses and the improvement of severity of disability (14–16).

RTX is a monoclonal antibody against CD20 on the surface of B cells that was approved initially to treat non-Hodgkin lymphoma (17). Given the pathogenesis of AQP4-IgG mediated humoral immunity in NMOSD, B cell depletion by RTX off-label use has been considered as a possible treatment approach and shows good efficacy, and thus has been highly recommended as first-line therapy (18–20). However, there are no consensus on the protocols of RTX induction therapy and re-treatment, including optimal RTX dosage and timing of therapy initiation. In the majority of previous studies, patients received intravenous infusion of 1 g RTX twice 2 weeks apart or 375 mg/m^2^ once weekly for 4 weeks as induction therapy, and maintenance reinfusions were administered mainly based on circulating B-cell subset percentages or counts or at a pre-specified fixed interval (19). However, the timing for initiating RTX therapy remains undetermined. Additionally, long-term high-dose RTX therapy may increase the risk of adverse effects of immunosuppression especially fatal consequences (11, 19, 21). The associated costs and treatment compliance of long-term therapy should also be taken into consideration in the majority of NMOSD patients. Thus, as alternatives of conventional high-dose RTX therapy, any effective treatment strategy that minimizes unnecessary exposure to the drug and allows significant cost savings and safety would be beneficial. Up till now, lower doses have been suggested, up to 100 mg RTX per infusion, to find the minimal effective dose in NMOSD and other autoimmune diseases (13, 22, 23). Herein, we addressed a novel lower-dose RTX (LD-RTX) strategy deriving from our real-world clinical practice and assessed its effectiveness and safety in treating NMOSD in a multicenter, open-label, self-controlled, prospective follow-up study conducted in the northwest of China.

## Methods

### Subjects and study design

This is a multicenter, open-label, self-controlled, prospective follow-up study registered at ClinicalTrials.gov (No.: NCT04256252). A total of eligible 108 patients with NMOSD were enrolled from January 2014 to May 2019. Inclusion criteria included: (1) age between 16 and 75 years old, (2) fulfilling 2006 diagnostic criteria for NMO (24) or 2015 diagnostic criteria for NMOSD (25), (3) at least two relapses in past 2 years and/or at least one attack or relapse in past one years, (4) willingness to enrollment of this study and disease-related assessments, and (5) negative pregnancy tests for female subjects prior to inclusion, and effective contraception for all subjects during the study period. The following exclusion criteria were applied: (1) use of other immunosuppressive agents or discontinuation for less than three months, (2) white blood cell < 3×10^9^/L, neutrophil < 1.5×10^9^/L, hemoglobin < 85 g/L, or platelet < 80×10^9^/L, (3) coexisting active liver disease or persistent transaminases elevation, (4) presence of serious blood, kidney, cardiovascular, and endocrine diseases, serious infection, or history of malignancies, (5) other chronic active immune diseases or stable conditions but requiring immunosuppressivr agents or glucocorticoids, (6) pregnant or lactating subjects and those with family planning, and (7) allergy to rituximab and its other components. The present study was approved by the Ethics Committee of Tangdu Hospital, Air Force Medical University (approval No. 2014120), and informed consent was obtained from all subjects or their legal representatives before LD-RTX therapy initiation.

### Treatment protocol

The LD-RTX therapy protocol consisted of induction therapy and maintenance therapy. RTX was administered at a dose of 100 mg once weekly for 3 weeks as induction therapy. Maintenance therapy was reinfusions of RTX at 100 mg once every 6 months according to the percentages of circulating B cell subsets and patient’s preference. Rescue therapy for relapses was intravenous methylprednisolone 500-1000 mg for 5 consecutive days, and then oral prednisone was initiated at 0.6-1.0 mg/kg and decreased gradually over approximately 3 months. An additional cycle of RTX induction therapy was administered following intravenous methylprednisolone therapy for relapses. Before each RTX infusion, the patient received 0.3 g of oral Ibuprofen and 10 mg of intravenous dexamethasone to prevent possible flu-like symptoms and infusion-related allergic reactions. The percentages of circulating B cell subsets in peripheral blood mononuclear cells (PBMCs) were assessed by flow cytometry before and after LD-RTX induction therapy, and then every 6 months. B-cell depletion was defined as a percentage of CD19^+^ B cells lower than 1% and CD19^+^CD27^+^ memory B cells lower than 0.05%. Once B cell repopulation (percentages of CD19^+^ B cells > 1% and/or memory B cells > 0.05%) occurred at the pre-specified time points, a maintenance reinfusion of LD-RTX would be recommended.

### Efficacy and safety assessments

All the subjects were treated with LD-RTX for at least 12 months. The primary outcome measure for efficacy assessments was annualized relapse rate (ARR) before and after LD-RTX therapy. Clinical relapses were defined as the occurrence of new neurological symptoms or the worsening of previous symptoms maintaining more than 24 hours, with an increase of DESS score by at least 0.5 points. The change of EDSS scores prior to and after LD-RTX therapy was used as the secondary outcome measure, and the lesions on spinal cord MRI were evaluated before and after LD-RTX therapy. In addition, the safety profile was assessed by recording all adverse events related to RTX use. During this study period, EDSS scores were evaluated by two qualified neurologists together.

### Statistical analyses

Categorical data are shown as number with percentage and continuous data as median with interquartile range (IQR). The chi-square test or Fisher’s exact test was used for categorical data to compare the inter-group differences, and Wilcoxon rank sum test or Student’s *t* test was used for inter-group comparisons of continuous data with skew or normal distribution. The changes of ARR, EDSS and spinal cord lesions before and after LD-RTX therapy were analyzed by the Wilcoxon matched-pairs signed rank test. As previously suggested (14), pre-RTX ARR could be calculated only when disease duration prior to LD-RTX therapy initiation lasted for at least 6 months to avoid the potential overestimation. For ARR comparison, only patients with ARR values of both pre- and post-RTX were included. The Kaplan-Meier survival curve was adopted to analyze the cumulative risk of relapses during pre- versus post-RTX periods and in early versus delayed RTX therapy groups, and inter-group differences were compared by the Mantel-Cox log-rank test. All statistical analyses were performed using the SPSS 23.0 software, Graphpad Prism 7 and R software version 4.2.2 for Windows. Statistical significance was set at the level of *p* < 0.05.

## Results

### Patient characteristics

**Figure 1:**
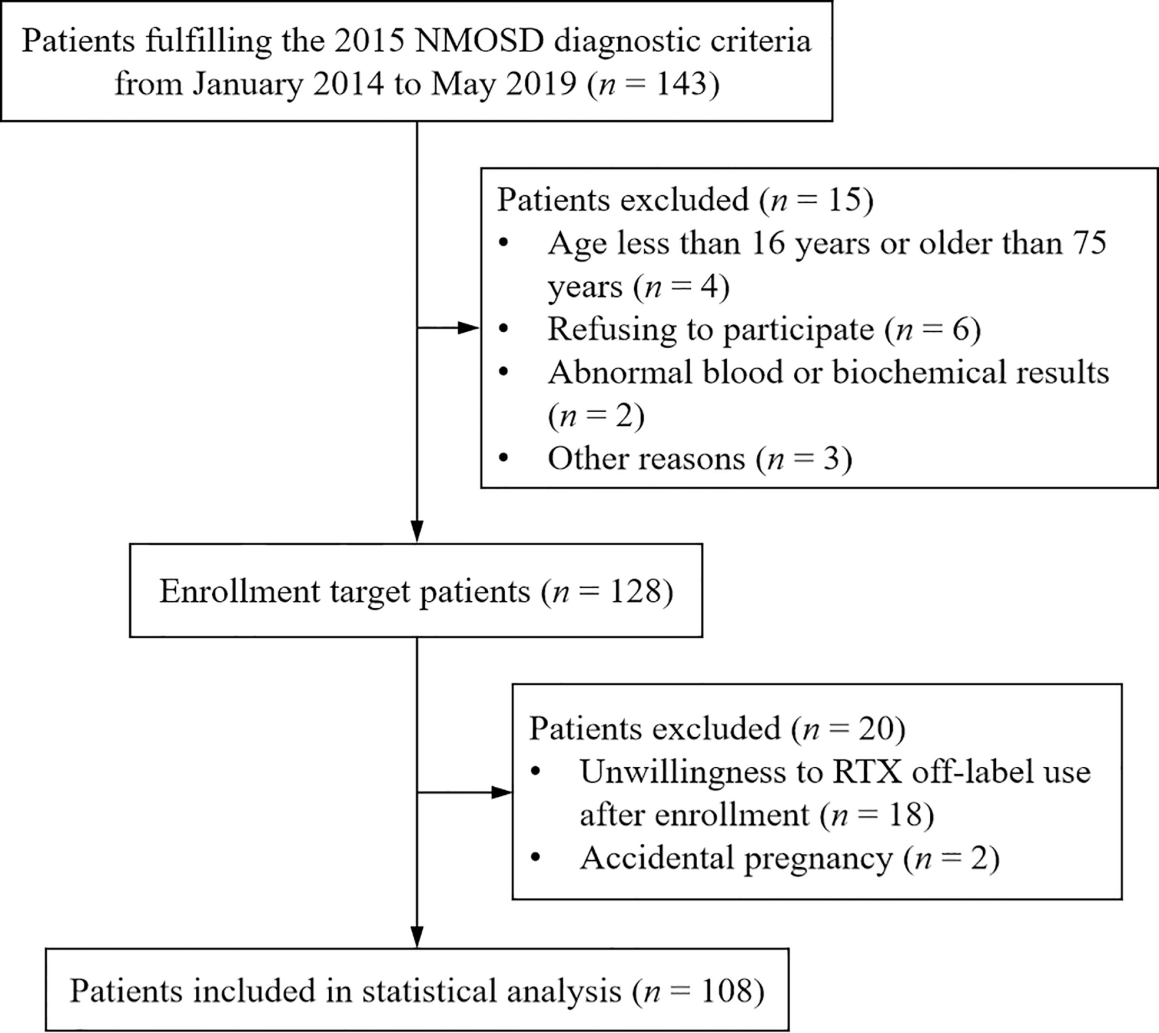
Flowchart of NMOSD patient enrollment in this study.

**Figure 2:**
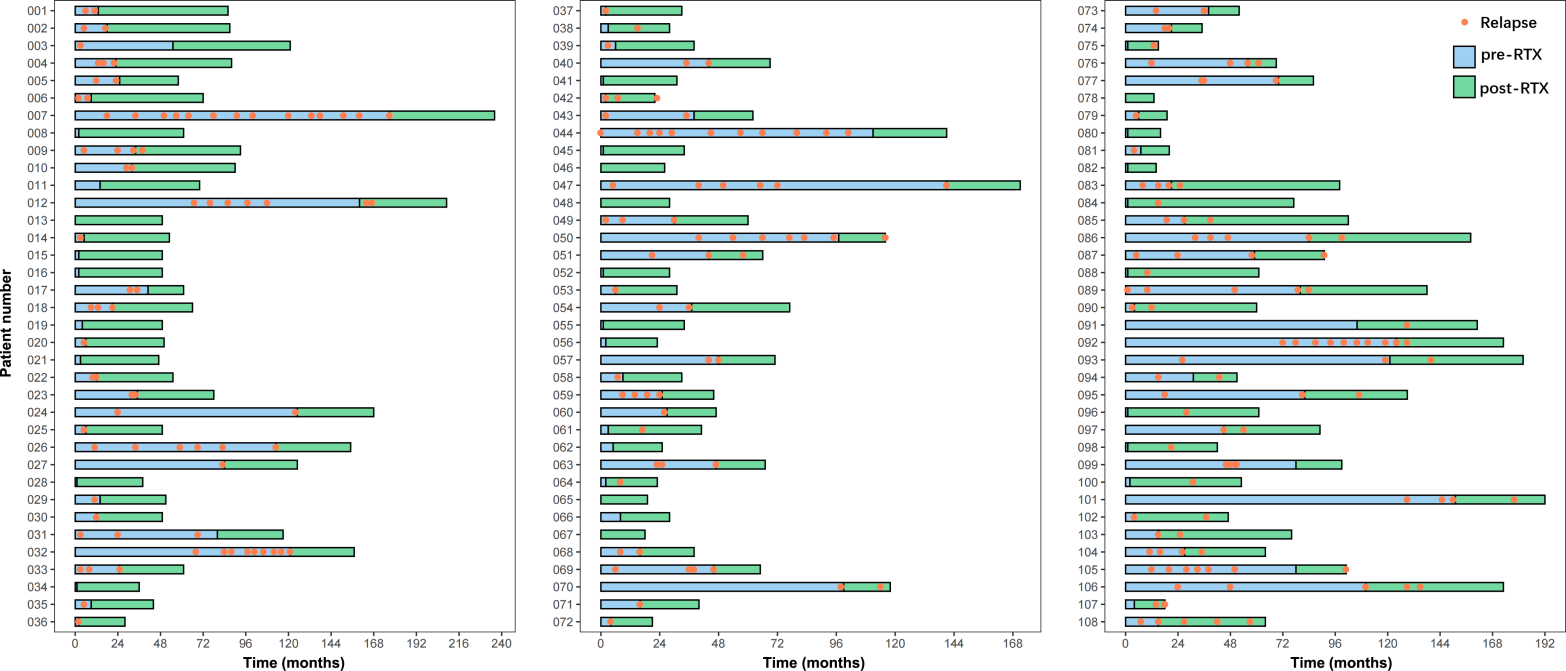
The detailed information on relapses and LD-RTX therapy throughout the disease course of all 108 patients. “0” on the x-axis represents the disease onset.

A total of 108 eligible patients diagnosed with NMOSD were eventually enrolled in the present study, with the enrollment flowchart shown in **Figure 1**. Of them, 11 patients enrolled between January 2014 and November 2015 according to 2006 NMO diagnostic criteria initially also fulfilled 2015 NMOSD diagnostic criteria. **Table 1** illustrates the baseline characteristics of all the patients, including 96 females and 12 males. AQP4-IgG was detected positive in sera from 92 patients, and the other 15 cases had negative results and one case with the result not available, with the comparison of clinical characteristics were revealed in **Table 1**. Overall, the median age at onset was 39.5 (IQR, 28.3–51.0) years and the median interval from disease onset to LD-RTX therapy initiation was 15.0 (2.0–46.8) months. Myelitis was the most common phenotype of the first attack with an incidence rate of 51.9%, followed by optic neuritis (31.5%). The clinical characteristics of patients who had experienced one attack or at least 2 attacks prior to LD-RTX therapy initiation were compared and shown in **Table 2**. LD-RTX therapy was initiated in 80 (74.1%) patients immediately after intravenous methylprednisolone rescue therapy for acute attacks, whereas other 28 (25.9%) initiated at the remission stage. All the patients had received immunotherapy prior to RTX use. Of them, 107 (99.1%) has ever treated with steroids, 13 (12.0%) with intravenous immunoglobulin, and 21 (19.4%) with other immunosuppressants including mycophenolate mofetil (MMF), azathioprine (AZA), ciclosporin A (CYC), and cyclophosphamide (CTX). Of note, these immunosuppressive agents had been discontinued for at least 3 months prior to LD-RTX therapy initiation. B-cell depletion was achieved in all subjects after LD-RTX induction therapy. The detailed data on relapses and LD-RTX therapy throughout disease course of all the 108 patients was shown in **Figure 2**. In total, 35 (32.4%) patients experienced at least one relapse during the post-RTX period, and the occurrence of each relapse and its time relevance to the last reinfusion of RTX was depicted in **Supplemental Figure 1**. Moreover, the comparison of clinical characteristics of patients with relapses or not during the post-RTX period was revealed in **Supplemental Table 1**. Of the 35 patients who experienced post-RTX relapses, 4 had switched from RTX to AZA or MMF till the last follow-up. In addition, three patients had been enrolled in other clinical trials but not due to dissatisfaction with the efficacy of LD-RTX therapy. One patient had died of depression not related to NMOSD.

**Table 1:** Clinical characteristics of 108 NMOSD patients enrolled in this study.

Characteristic	Total	AQP4-IgG positive (*N*=92)	AQP4-IgG negative or not available (*N*=16)	*p* value
Female, *n* (%)	96 (88.9)	83 (90.2)	13 (81.3)	0.381
Age at onset, year, median (IQR)	39.5 (28.3–51.0)	40.5 (28.3–51.0)	37.5 (28.5–49.0)	0.747
Clinical phenotype of the first attack				
Myelitis, *n* (%)	56 (51.9)	49 (53.3)	7 (43.8)	0.591
Optic neuritis, *n* (%)	34 (31.5)	28 (30.4)	6 (37.5)	0.771
Area postrema syndrome, *n* (%)	12 (11.1)	10 (10.9)	2 (12.5)	1.000
Acute brainstem syndrome, *n* (%)	3 (2.8)	2 (2.2)	1 (6.3)	0.385
Cerebral syndrome, *n* (%)	2 (1.9)	2 (2.2)	0 (0)	1.000
Myelitis and optic neuritis, *n* (%)	1 (0.9)	1 (1.1)	0 (0)	1.000
Concomitant autoimmune diseases, *n* (%)	3 (2.8)	3 (3.3)	0 (0)	1.000
Other immunosuppressants use pre-RTX, *n* (%)	21.0 (19.4)	17 (18.5)	4 (25.0)	0.509
Disease duration pre-RTX, month, median (IQR)	15.0 (2.0–46.8)	12.5 (2.0–46.8)	29.5 (13.0–52.3)	0.064
Disease duration post-RTX, month, median (IQR)	35.5 (22.0–48.8)	35.5 (21.0–47.8)	35.5 (24.0–56.5)	0.772
Number of RTX cycle after induction, median (IQR)	4 (3–7)	4 (3–7)	4 (2–7)	0.770
ARR pre-RTX, median (IQR)	1.1 (0.8–2.0)	1.0 (0.8–1.7)	1.7 (0.8–2.4)	0.261
ARR post-RTX, median (IQR)	0 (0–0.2)	0 (0–0.2)	0 (0–0)	0.064
EDSS pre-RTX, median (IQR)	3.5 (2.5–4.0)	3.5 (2.5–4.4)	2.5 (2.0–3.4)	0.034
EDSS post-RTX, median (IQR)	2.0 (1.0–3.0)	2.0 (1.0–3.5)	1.0 (1.0–1.9)	0.011
Side effect, *n* (%)	22 (20.4)	16 (17.4)	6 (37.5)	0.090

ARR, annualized relapse rate; AQP4, aquaporin-4; EDSS, expanded disability status scale; IQR, interquartile range; NMOSD, neuromyelitis optica spectrum disorder; RTX, rituximab.

**Table 2:** Comparison of clinical characteristics of patients according to the number of attacks prior to LD-RTX therapy initiation.

Characteristic	One attack (*N*=33)	At least two attacks (*N*=75)	*p* value
Female, *n* (%)	29 (87.9)	67 (89.3)	1.000
Age at onset, year, median (IQR)	49.0 (31.0–53.0)	38.0 (27.0–49.0)	0.024
Serum AQP4-IgG positive, *n* (%)	33 (100.0)	59 (78.7)	0.002
Clinical phenotype of the first attack			
Myelitis, *n* (%)	19 (57.6)	37 (49.3)	0.531
Optic neuritis, *n* (%)	8 (24.2)	26 (34.7)	0.370
Area postrema syndrome, *n* (%)	4 (12.1)	8 (10.7)	1.000
Acute brainstem syndrome, *n* (%)	1 (3.0)	2 (2.7)	1.000
Cerebral syndrome, *n* (%)	0 (0)	2 (2.7)	1.000
Myelitis and optic neuritis, *n* (%)	1 (3.0)	0 (0)	0.306
Number of attacks pre-RTX, median (IQR)	1 (1–1)	3 (2–4)	NA
Number of relapses post-RTX, median (IQR)	0 (0–1)	0 (0–1)	0.767
Disease duration pre-RTX, month, median (IQR)	1.0 (1.0–2.5)	32.0 (13.0–80.0)	<0.001
Disease duration post-RTX, month, median (IQR)	33.0 (20.0–47.0)	37.0 (23.0–49.0)	0.401
ARR pre-RTX, median (IQR)	NA	1.1 (0.8–2.1)	NA
ARR post-RTX, median (IQR)	0 (0–0.2)	0 (0–0.2)	0.785
EDSS pre-RTX, median (IQR)	3.0 (2.5–4.0)	3.5 (2.0–4.5)	0.752
EDSS post-RTX, median (IQR)	2.0 (1.0–2.0)	2.0 (1.0–3.5)	0.870
Side effect, *n* (%)	8 (24.2)	14 (18.7)	0.605

ARR, annualized relapse rate; AQP4, aquaporin-4; EDSS, expanded disability status scale; IQR, interquartile range; RTX, rituximab. NA, not applicable.

### Effects of LD-RTX therapy on ARR and EDSS scores

**Figure 3:**
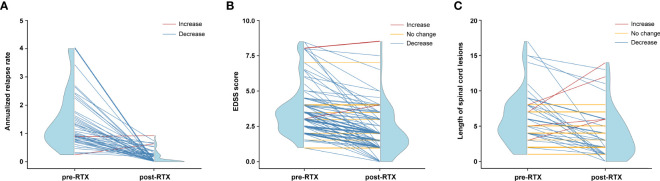
Efficacy assessments of LD-RTX therapy in each NMOSD patient. **(A)** Changes in annualized relapse rate (ARR) after therapy compared to before therapy (*n* = 70). Thirty-eight patients were not included due to a disease duration of less than 6 months prior to the first RTX infusion. Pre-RTX indicates the period from disease onset to the initiation of LD-RTX therapy, and post-RTX indicates the period from the initiation of LD-RTX therapy to the last follow-up. **(B)** Changes in EDSS score after therapy compared to before therapy (*n* = 108). **(C)** Changes in length of spinal cord lesions after therapy compared to before therapy (*n* = 53). The red line indicates an increased tendency, the yellow indicates no change and the blue indicates an decreased tendency. Blue half violin shows the distribution of efficacy measures at each time point. For **(B, C)**, pre-RTX indicates the time point of disease onset and post-RTX indicates the time point of the last follow-up.

The efficacy of LD-RTX therapy was evaluated mainly by the changes in ARR and EDSS score during a median post-RTX follow-up period of 35.5 (22.0–48.8) months. Overall, a dramatic decrease of median ARR was achieved at the end of this study compared with that prior to RTX therapy (0 [0–0.2] versus 1.1 [0.8–2.0], *Z* -7.196, *p* < 0.001). It was of note that the overwhelming majority of the subjects (97.1%, 68/70) had a reduction in their ARRs (**Figure 3A**) and 73 (67.6%) were relapse-free. Similar to ARR, LD-RTX therapy also led to a significant decrease of EDSS score, with the median score from 3.5 (2.5–4.0) before RTX therapy to 2.0 (1.0–3.0) after therapy (*Z* -8.320, *p* < 0.001). Specifically, the EDSS score decreased in 93 (86.1%) cases and remained unchanged in 12 (11.1%) cases (**Figure 3B**).

**Figure 4:**
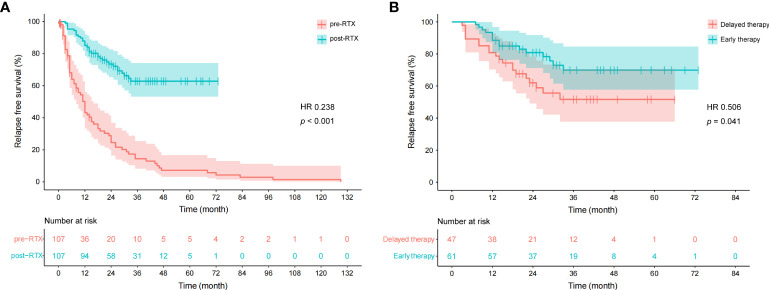
Cumulative survivals without relapse over time (months) as depicted by the Kaplan-Meier curve. **(A)** Comparison between pre- and post-RTX periods. One patient was not included due to the time from disease onset to the first relapse not available. “0” on the x-axis represents the disease onset in the pre-RTX group and the initiation of LD-RTX therapy in the post-RTX group. **(B)** Comparison between early therapy and delayed RTX therapy. “0” on the x-axis represents the initiation of LD-RTX therapy. Patients who experienced the first relapse from the pre-specified time point were censored, as presented by narrow vertical lines.

The median time to first relapse was 11.0 (4.8–24.0) months for the patients who experienced at least one relapse during the pre-RTX period, while the value was 12.0 (7.8–19.2) months for those during the post-RTX period (inter-group comparison, *p* = 0.501). Moreover, the median time to the first relapse was 14.0 (10.0–27.0) months for patients who experienced at least one relapse on early therapy initiated within 24 months after disease onset and 13.0 (4.0–18.0) months for those on delayed therapy (inter-group comparison, *p* = 0.316). We used the Kaplan-Meier survival curves to analyze the cumulative risk of developing relapses in all patients. The cumulative risk during the post-RTX period was significantly lower than that during the pre-RTX period (hazard ratio [HR] 0.238, 95%CI 0.160–0.356, *p* < 0.001; **Figure 4A**). As illustrated in **Figure 4B**, early RTX therapy was associated with a decreased cumulative risk compared to delayed therapy (HR 0.506, 95%CI 0.258–0.994, *p* = 0.041).

### Effects of LD-RTX therapy on spinal cord lesion segments

As an additional index of therapeutic efficacy, the changes in spinal cord lesions were compared in 53 patients who had available pre- and post-RTX MRI results. The median length of the lesion segments was 5.0 (4.0–8.0) before LD-RTX therapy and dropped to 3.0 (1.0–6.0) after therapy (*Z* -3.984, *p* < 0.001). As shown in **Figure 3C**, the decrease in the length of spinal cord lesions occurred in 31 (58.5%) cases and lesions disappeared completely in 6 (11.3%) cases. Although no changes in spinal cord segments were observed after RTX therapy in the remaining 16 (30.2%) cases, an obvious reduction of lesion volume was confirmed in these patients (data not shown).

### Circulating B cell monitoring

**Figure 5:**
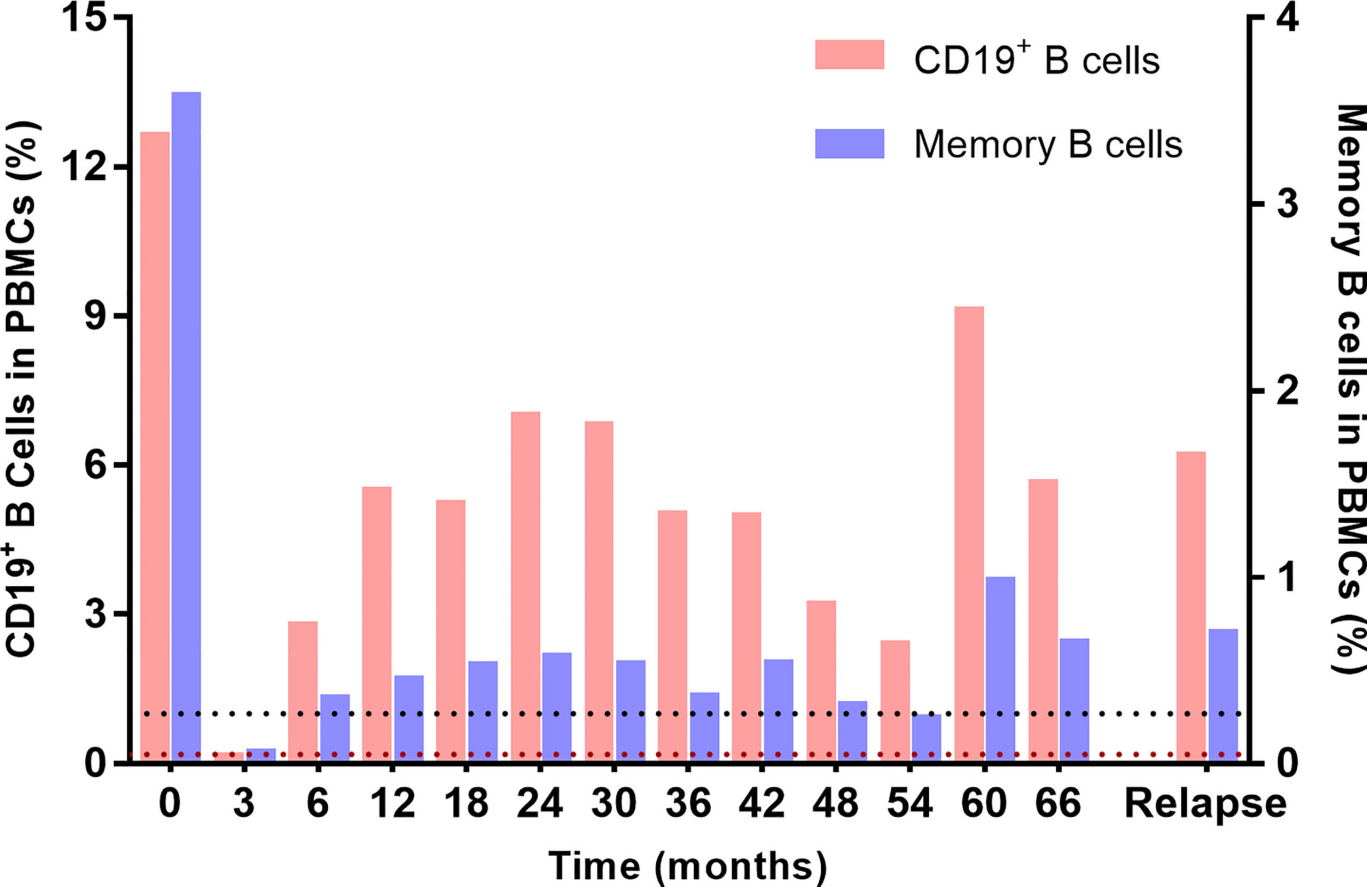
Dynamic monitoring of circulating CD19^+^ B cells and CD19^+^CD27^+^ memory B cells in PBMCs. The black dotted line represents the cut-off percentage of CD19^+^ B cells of 1%, and the brown dotted line represents the cut-off percentage of memory cells of 0.05%.

Flow cytometry was performed to monitor the dynamics of circulating CD19^+^ B cell and CD19^+^CD27^+^ memory B cell subsets throughout LD-RTX therapy period. LD-RTX induction therapy led to B-cell depletion in all patients and this effect still remained at 3 months after induction. At 6 months after induction and thereafter, the median percentages of these two cell subsets in total PBMCs always maintained above the cut-off value of B-cell depletion (**Figure 5**), and similar percentages were detected when post-RTX clinical relapses occurred.

### Safety profile of LD-RTX therapy

During the study period, 22 (20.4%) of 108 patients experienced RTX-related side effects, including infusion-related reactions and other adverse events. As shown in **Table 3**, skin rash was the most common infusion-related reaction occurring in 7 (31.8%) of the 22 patients, followed by flu-like symptoms (27.3%, 6/22), skin pruritus (18.2%, 4/22), sweating (13.6%, 3/22), palpitation (4.5%, 1/22) and laryngeal edema (4.5%, 1/22). All the side effects were mild and transient and could be rapidly relieved by lowering the infusion rate or by anti-allergic therapy. Other adverse events included alopecia (36.4%, 8/22), fatigue (9.1%, 2/22), muscle aches (9.1%, 2/22), urinary infection (4.5%, 1/22) and hemoglobin drop (4.5%, 1/22). No serious side effects such as tumors, progressive multifocal leukoencephalopathy, and serious infections were observed during the study period.

**Table 3:** Distribution of LD-RTX-related side effects in 22 NMOSD patients.

	Patients with side effects, *n* (%)
Infusion-related reactions	
Skin rash	7 (31.8)
Flu-like symptoms	6 (27.3)
Skin pruritus	4 (18.2)
Sweating	3 (13.6)
Palpitation	1 (4.5)
Laryngeal edema	1 (4.5)
Other adverse events	
Alopecia	8 (36.4)
Fatigue	2 (9.1)
Muscle aches	2 (9.1)
Urinary infection	1 (4.5)
Hemoglobin drop	1 (4.5)

LD-RTX, low-dose rituximab; NMOSD, neuromyelitis optica spectrum disorder.

## Discussion

Rituximab (RTX) has been well recommended as first-line therapy to prevent relapses of NMOSD. However, there is no consensus on RTX dosage regimen due to the fact of off-label use and the lack of large-scale prospective randomized controlled trials. This study was led by Tangdu Hospital of Air Force Medical University, where a dose of 100 mg RTX once weekly for 3 weeks as induction therapy and reinfusions of 100 mg RTX every 6 months as maintenance therapy are the typical treatment approach for NMOSD patients. Based on the pilot satisfactory outcome of this strategy in our single center, we conducted this multicenter, open-label, self-controlled, prospective follow-up study in the northwest of China to clarify the effectiveness and safety of this LD-RTX strategy in treating NMOSD. The key findings include the following: (1) our LD-RTX strategy is demonstrated to be effective based on the significant decrease of ARR, EDSS score and length of spinal cord lesions after RTX therapy; (2) the satisfactory safety profile of our regimen is also confirmed based on a low risk of developing RTX-related side effects and a low switch rate to other immunosuppressants; and (3) the dramatic decrease of cumulative risk of relapses during post- versus pre-RTX therapy periods and on early versus delayed therapy reinforces the necessity and importance of initiating LD-RTX therapy as early as possible.

The efficacy of RTX in treating NMOSD has been widely demonstrated since the first report by Cree et al. in 2005 (12). However, the majority of prior studies conducted in Caucasian population have adopted the high dose of RTX induction regimen similar to that used in B cell lymphoma, i.e., 375 mg/m^2^ once weekly for 4 consecutive weeks or 1000 mg twice 2 weeks apart. The maintenance reinfusions (500-1000 mg/cycle) were administered every 6 to 9 months according to clinical functional status and patient’s choice (26) or depending on circulating B cell repopulation. Besides the economic costs of this high-dose regimen, the prescription should be given with caution due to the potentially serious adverse effects including death (19, 21). In such a perspective, low-dose strategies would be of great promise in the context of ensuring efficacy and safety. During the last decade, several groups from China have attempted various modified dosages of RTX strategies for NMOSD and achieved a good efficacy and safety profile, exhibiting a better efficacy than other immunosuppressive agents such as AZA or MMF (13, 27, 28). In these retrospective studies of small sample sizes, the RTX induction regimen included 100 mg once weekly for 3-4 consecutive weeks, and then the percentages of circulating CD19^+^ B cells and/or CD19^+^CD27^+^ memory B cells were monitored every 4-12 weeks. The maintenance infusion was restarted whenever B cells were repopulated. In contrast, a lower dose of RTX strategy was applied in this study than those in any prior studies, including 300 mg of induction dose and then repeated maintenance reinfusions of 100 mg at a fixed 6-month interval. Encouragingly, our LD-RTX strategy maintained good efficacy over a median of 35.5 months, as manifested by the marked reduction of median ARR by 100%, median EDSS score by 43%, and median length of spinal cord lesions by 40%. More notably, 67.6% of patients were relapse-free, 86.1% had improved EDSS scores and 58.5% had reduced or disappeared spinal cord lesion segments, which is similar to those of other studies assessing the effectiveness of low- or standard-dose RTX strategies in treating NMOSD (29–31). In our opinion, the lower dose of RTX strategy applied in this study would be a promising option for treating NMOSD. Additionally, our data provide insights into optimal RTX dosing exploration in the future.

To date, the timing of initiating RTX therapy in NMOSD patients remains undetermined. In a recent practical guideline of the NOMADMUS group from France, an expert consensus was obtained for recommending no window between the end of rescue therapy for acute attacks (i.e., high doses of steroids and/or plasma exchanges) and RTX therapy initiation (32). The view is well followed in this study where 80 (74.1%) patients started LD-RTX therapy immediately after acute attack treatment, which simulates the real-world setting in clinical practice. Another retrospective study provides a novel clue for early RTX use based on the finding that RTX therapy initiated within 24 months of NMO onset resulted in a more dramatic reduction of ARR, although the same dosages of RTX initiated 24 months later also achieved a significant ARR drop compared with before treatment (20). In this study, 61 patients received early LD-RTX therapy and more than half (54.1%, 33/61) of the cases were first-onset. The concept of starting RTX therapy earlier is further reinforced since the cumulative risk of relapses in the patients receiving early LD-RTX therapy significantly decreased compared to those receiving delayed therapy. Although the median time to the first relapse was not markedly prolonged during post- versus pre-RTX therapy periods and on early versus delayed therapy, a dramatic decrease of cumulative risk of relapses gives sufficient support to the use of RTX as early as possible and as sequential therapy following acute attack treatment.

Regarding the timing of maintenance RTX reinfusion, there is an absence of a standardized protocol. The majority of previous studies monitored the count and/or percentage of circulating CD19^+^ B cells, especially CD19^+^CD27^+^ memory cells at varying time intervals. A widely accepted cut-off of B-cell depletion are the percentage of CD19^+^ B cells lower than 1% (22), or that of memory B cells lower than 0.05% in the initial two years and 0.1% thereafter (18). RTX reinfusions were administered whenever B cells were repopulated, even though the dosage of RTX varied from one to another (13, 18, 20, 33, 34). Greenberg et al. (22) compared the time to B-cell repopulation (defined as a CD19 percentage of 2% or greater) after different RTX doses and found that 1000 mg per dose yielded more prolonged B-cell depletion than 100 mg per dose (184 ± 72 days versus 99 ± 36 days), which may provide support to more frequent reinfusion when low RTX dosing strategies were applied (13, 27, 28, 35, 36). Although the association of clinical relapses with B-cell repopulation has been well determined to date, it is of note that we have observed that many patients with B-cell repopulation maintained a long-term relapse-free condition in real-world clinical practice. Similar findings come from a recent study in which 2 NMOSD patients receiving low-dose RTX therapy maintained a relapse-free condition at the majority of time points when B-cell repopulated but not receiving reinfusions, raising the question of whether the low threshold of B cells is an obligatory monitoring indicator of repeated RTX reinfusions (37). In this study, a fixed 6-month interval of RTX reinfusion was determined based on our clinical experience and it was longer than those of other low-dose RTX strategies (13, 27, 28, 35, 36). Encouragingly, our strategy with a longer time interval of reinfusion is sufficient to maintain a clinical remission status, as demonstrated by 67.6% relapse-free patients at the end of the study. Thus, our results reopen the issue of the optimal cut-off level of B cells for repeated RTX reinfusions which required future investigation.

The safety and tolerability of our LD-RTX strategy are well established. The frequency of side effects in our study was lower than the conventional dose strategies (18, 34, 38) but similar to the low dose strategies (28, 36, 39). Consistent with these prior studies, the most common were infusion-related reactions. It is of note that no serious adverse events were recorded and no patients discontinued RTX treatment because of side effects related to RTX use. Meanwhile, no increment of the incidence rate of side effects were determined with the extension of therapy duration. Considering that long-term high-dose RTX may be accompanied by the increased risk of adverse effects of immunosuppression especially fatal outcomes (11, 19, 21), our strategy with a lower cumulative dose of RTX might become a better treatment option guaranteeing both efficacy and safety.

Our research has several limitations. Firstly, it was not designed in a randomized, double-blind manner. Thus, the control group was absent and it would inevitably limit the level of evidence from our data. Secondly, a high proportion (35.2%, 38/108) of patients have a disease duration of less than 6 months prior to the first RTX infusion. As a result, these patients were not included when comparing the ARR before and after RTX therapy. It was sufficient to avoid the potential overestimation, but future investigation with valid data for all enrolled subjects is warranted to achieve reliable conclusions. Thirdly, we notice that 74.1% of patients had no therapeutic window between the end of rescue therapy for acute attack and LD-RTX therapy initiation. Although this reflects real-world clinical situations, RTX therapy following glucocorticoids might have affected the post-RTX evaluations of EDSS score and length of spinal cord lesions. Thus, these two are only taken as the secondary outcome measures in the present study. Future prospective, well-designed investigations are required to validate the conclusions derived from our study.

In conclusion, a novel lower-dose RTX therapy strategy is addressed and new attempts in terms of RTX initiation and reinfusion timing are made in this study. Our LD-RTX therapy significantly reduces clinical relapses and disability and would be of great promise in the context of ensuring both efficacy and safety.

## Data availability statement

The raw data supporting the conclusions of this article will be made available by the authors, without undue reservation.

## Ethics statement

The studies involving human participants were reviewed and approved by Ethics Committee of Tangdu Hospital, Air Force Medical University. Written informed consent to participate in this study was provided by the participants’ legal guardian/next of kin.

## Author contributions

HL and JG conceived and designed the protocol. ZQL, ZBL, JW, ZX, TL, CM, SZ and MB collected the data. DZ, KR and JL wrote the manuscript; DZ and KR analyzed the data. HL and JG critically revised the manuscript. All authors contributed to the article and approved the submitted version.

## References

[1] Jarius S Paul F Weinshenker BG Levy M Kim HJ Wildemann B. Neuromyelitis optica. Nat Rev Dis Primers (2020) 6(1):85. doi: 10.1038/s41572-020-0214-9 33093467

[2] Lennon VA Wingerchuk DM Kryzer TJ Pittock SJ Lucchinetti CF Fujihara K. A serum autoantibody marker of neuromyelitis optica: distinction from multiple sclerosis. Lancet (2004) 364(9451):2106–12. doi: 10.1016/S0140-6736(04)17551-X 15589308

[3] Bizzoco E Lolli F Repice AM Hakiki B Falcini M Barilaro A. Prevalence of neuromyelitis optica spectrum disorder and phenotype distribution. J Neurol (2009) 256(11):1891–8. doi: 10.1007/s00415-009-5171-x 19479168

[4] Sherman E Han MH. Acute and chronic management of neuromyelitis optica spectrum disorder. Curr Treat Options Neurol (2015) 17(11):48. doi: 10.1007/s11940-015-0378-x 26433388 PMC4592697

[5] Yamamura T Kleiter I Fujihara K Palace J Greenberg B Zakrzewska-Pniewska B. Trial of satralizumab in neuromyelitis optica spectrum disorder. N Engl J Med (2019) 381(22):2114–24. doi: 10.1056/NEJMoa1901747 31774956

[6] Traboulsee A Greenberg BM Bennett JL Szczechowski L Fox E Shkrobot S. Safety and efficacy of satralizumab monotherapy in neuromyelitis optica spectrum disorder: a randomised, double-blind, multicentre, placebo-controlled phase 3 trial. Lancet Neurol (2020) 19(5):402–12. doi: 10.1016/S1474-4422(20)30078-8 PMC7935419 32333898

[7] Cree BAC Bennett JL Kim HJ Weinshenker BG Pittock SJ Wingerchuk DM. Inebilizumab for the treatment of neuromyelitis optica spectrum disorder (N-MOmentum): a double-blind, randomised placebo-controlled phase 2/3 trial. Lancet (2019) 394(10206):1352–63. doi: 10.1016/S0140-6736(19)31817-3 31495497

[8] Pittock SJ Berthele A Fujihara K Kim HJ Levy M Palace J. Eculizumab in aquaporin-4-positive neuromyelitis optica spectrum disorder. N Engl J Med (2019) 381(7):614–25. doi: 10.1056/NEJMoa1900866 31050279

[9] Espiritu AI Pasco PMD. Efficacy and tolerability of azathioprine for neuromyelitis optica spectrum disorder: A systematic review and meta-analysis. Mult Scler Relat Disord (2019) 33:22–32. doi: 10.1016/j.msard.2019.05.011 31136907

[10] Songwisit S Kosiyakul P Jitprapaikulsan J Prayoonwiwat N Ungprasert P Siritho S. Efficacy and safety of mycophenolate mofetil therapy in neuromyelitis optica spectrum disorders: a systematic review and meta-analysis. Sci Rep (2020) 10(1):16727. doi: 10.1038/s41598-020-73882-8 33028926 PMC7541495

[11] Jacob A Weinshenker BG Violich I McLinskey N Krupp L Fox RJ. Treatment of neuromyelitis optica with rituximab: retrospective analysis of 25 patients. Arch Neurol (2008) 65(11):1443–8. doi: 10.1001/archneur.65.11.noc80069 18779415

[12] Cree BA Lamb S Morgan K Chen A Waubant E Genain C. An open label study of the effects of rituximab in neuromyelitis optica. Neurology (2005) 64(7):1270–2. doi: 10.1212/01.WNL.0000159399.81861.D5 15824362

[13] Yang CS Yang L Li T Zhang DQ Jin WN Li MS. Responsiveness to reduced dosage of rituximab in Chinese patients with neuromyelitis optica. Neurology (2013) 81(8):710–3. doi: 10.1212/WNL.0b013e3182a1aac7 PMC3776460 23884041

[14] Mealy MA Wingerchuk DM Palace J Greenberg BM Levy M. Comparison of relapse and treatment failure rates among patients with neuromyelitis optica: multicenter study of treatment efficacy. JAMA Neurol (2014) 71(3):324–30. doi: 10.1001/jamaneurol.2013.5699 24445513

[15] Torres J Pruitt A Balcer L Galetta S Markowitz C Dahodwala N. Analysis of the treatment of neuromyelitis optica. J Neurol Sci (2015) 351(1-2):31–5. doi: 10.1016/j.jns.2015.02.012 25727350

[16] Jeong IH Park B Kim SH Hyun JW Joo J Kim HJ. Comparative analysis of treatment outcomes in patients with neuromyelitis optica spectrum disorder using multifaceted endpoints. Mult Scler (2016) 22(3):329–39. doi: 10.1177/1352458515587752 26041804

[17] Grillo-López AJ. Rituximab: an insider’s historical perspective. Semin Oncol (2000) 27(6 Suppl 12):9–16. 11226006

[18] Kim SH Kim W Li XF Jung IJ Kim HJ. Repeated treatment with rituximab based on the assessment of peripheral circulating memory B cells in patients with relapsing neuromyelitis optica over 2 years. Arch Neurol (2011) 68(11):1412–20. doi: 10.1001/archneurol.2011.154 21747007

[19] Damato V Evoli A Iorio R. Efficacy and safety of rituximab therapy in neuromyelitis optica spectrum disorders: A systematic review and meta-analysis. JAMA Neurol (2016) 73(11):1342–8. doi: 10.1001/jamaneurol.2016.1637 27668357

[20] Zéphir H Bernard-Valnet R Lebrun C Outteryck O Audoin B Bourre B. Rituximab as first-line therapy in neuromyelitis optica: efficiency and tolerability. J Neurol (2015) 262(10):2329–35. doi: 10.1007/s00415-015-7852-y 26194198

[21] Etemadifar M Salari M Mirmosayyeb O Serati M Nikkhah R Askari M. Efficacy and safety of rituximab in neuromyelitis optica: Review of evidence. J Res Med Sci (2017) 22:18. doi: 10.4103/1735-1995.200275 28458709 PMC5367207

[22] Greenberg BM Graves D Remington G Hardeman P Mann M Karandikar N. Rituximab dosing and monitoring strategies in neuromyelitis optica patients: creating strategies for therapeutic success. Mult Scler (2012) 18(7):1022–6. doi: 10.1177/1352458511432896 22261118

[23] Zaja F Vianelli N Volpetti S Battista ML Defina M Palmieri S. Low-dose rituximab in adult patients with primary immune thrombocytopenia. Eur J Haematol (2010) 85(4):329–34. doi: 10.1111/j.1600-0609.2010.01486.x 20546023

[24] Wingerchuk DM Lennon VA Pittock SJ Lucchinetti CF Weinshenker BG. Revised diagnostic criteria for neuromyelitis optica. Neurology (2006) 66(10):1485–9. doi: 10.1212/01.wnl.0000216139.44259.74 16717206

[25] Wingerchuk DM Banwell B Bennett JL Cabre P Carroll W Chitnis T. International consensus diagnostic criteria for neuromyelitis optica spectrum disorders. Neurology (2015) 85(2):177–89. doi: 10.1212/WNL.0000000000001729 PMC4515040 26092914

[26] Ip VH Lau AY Au LW Fan FS Chan AY Mok VC. Rituximab reduces attacks in Chinese patients with neuromyelitis optica spectrum disorders. J Neurol Sci (2013) 324(1-2):38–9. doi: 10.1016/j.jns.2012.09.024 23040959

[27] Yang Y Wang CJ Wang BJ Zeng ZL Guo SG. Comparison of efficacy and tolerability of azathioprine, mycophenolate mofetil, and lower dosages of rituximab among patients with neuromyelitis optica spectrum disorder. J Neurol Sci (2018) 385:192–7. doi: 10.1016/j.jns.2017.12.034 29406904

[28] Zhang M Zhang C Bai P Xue H Wang G. Effectiveness of low dose of rituximab compared with azathioprine in Chinese patients with neuromyelitis optica: an over 2-year follow-up study. Acta Neurol Belg (2017) 117(3):695–702. doi: 10.1007/s13760-017-0795-6 28608315

[29] Jade JD Bansi S Singhal B. Rituximab in neuromyelitis optica spectrum disorders: our experience. Ann Indian Acad Neurol (2017) 20(3):229–32. doi: 10.4103/aian.AIAN_499_16 PMC5586117 28904454

[30] Kim W Kim SH Kim HJ. New insights into neuromyelitis optica. J Clin Neurol (2011) 7(3):115–27. doi: 10.3988/jcn.2011.7.3.115 PMC3212597 22087205

[31] Kim SH Jeong IH Hyun JW Joung A Jo HJ Hwang SH. Treatment outcomes with rituximab in 100 patients with neuromyelitis optica: influence of FCGR3A polymorphisms on the therapeutic response to rituximab. JAMA Neurol (2015) 72(9):989–95. doi: 10.1001/jamaneurol.2015.1276 26167726

[32] Ciron J Audoin B Bourre B Brassat D Durand-Dubief F Laplaud D. Recommendations for the use of Rituximab in neuromyelitis optica spectrum disorders. Rev Neurol (Paris) (2018) 174(4):255–64. doi: 10.1016/j.neurol.2017.11.005 29606320

[33] Kim SH Kim Y Kim G Park NY Jang HM Shin HJ. Less frequent rituximab retreatment maintains remission of neuromyelitis optica spectrum disorder, following long-term rituximab treatment. J Neurol Neurosurg Psychiatry (2019) 90(4):486–7. doi: 10.1136/jnnp-2018-318465 29929977

[34] Kim SH Huh SY Lee SJ Joung A Kim HJ. A 5-year follow-up of rituximab treatment in patients with neuromyelitis optica spectrum disorder. JAMA Neurol (2013) 70(9):1110–7. doi: 10.1001/jamaneurol.2013.3071 23897062

[35] Li T Zhang LJ Zhang QX Yang CS Zhang C Li YJ. Anti-Rituximab antibody in patients with NMOSDs treated with low dose Rituximab. J Neuroimmunol (2018) 316:107–11. doi: 10.1016/j.jneuroim.2017.12.021 29310942

[36] Zhao S Zhou H Xu Q Dai H Wei S. Efficacy of low-dose rituximab on neuromyelitis optica-associated optic neuritis. Front Neurol (2021) 12:637932. doi: 10.3389/fneur.2021.637932 34017301 PMC8129159

[37] Jing L Yu H Li J Li Y Liu X Li H. Is low threshold of B cells an obligatory monitoring indicator of repeated RTX infusion in the treatment of Neuromyelitis Optica spectrum disorder? - A report of two cases. Neuroimmunol Rep (2021) 1:100023. doi: 10.1016/j.nerep.2021.100023

[38] Bedi GS Brown AD Delgado SR Usmani N Lam BL Sheremata WA. Impact of rituximab on relapse rate and disability in neuromyelitis optica. Mult Scler (2011) 17(10):1225–30. doi: 10.1177/1352458511404586 21622594

[39] Xiao H Zeng W Li L Li L Cui Y Wang J. Retrospective observation of low-dose rituximab treatment in Chinese patients with neuromyelitis optica spectrum disorders in a real-world setting. Front Neurol (2020) 11:642. doi: 10.3389/fneur.2020.00642 32733365 PMC7358348

