# Supplementary material for: Rituximab at lower dose for neuromyelitis optica spectrum disorder: a multicenter, open-label, self-controlled, prospective follow-up study
Source: Front Immunol. 2023 Aug 8;14:1148632. doi: 10.3389/fimmu.2023.1148632 (PMC10442836; doi:10.3389/fimmu.2023.1148632)
Supplement: Supplementary file 1 [file DataSheet_1.docx]

**Supplemental data**


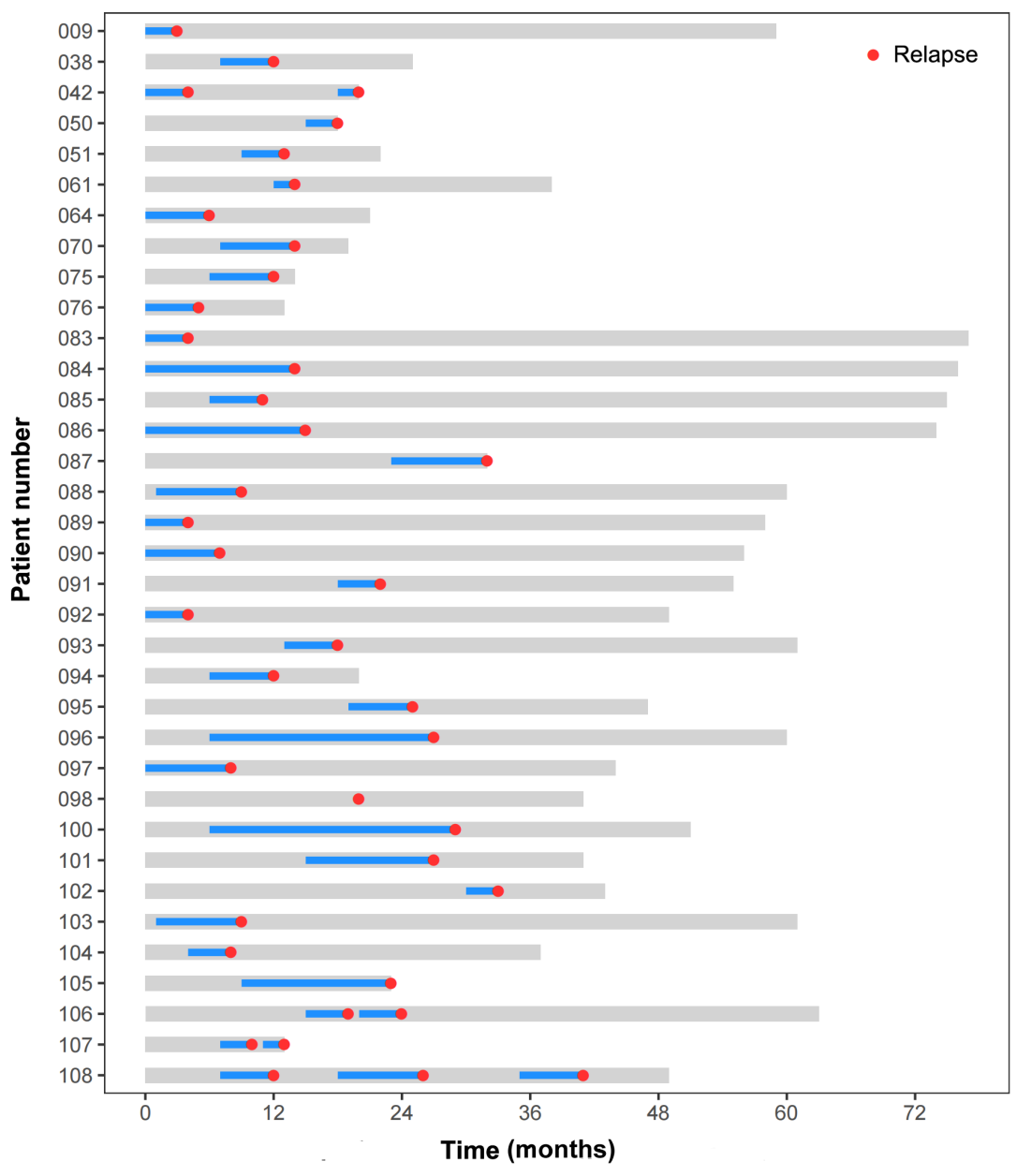


**Supplemental Figure 1:** Detailed characteristics of 35 NMOSD patients who experienced relapses during post-RTX period. “0” on the x-axis represents the LD-RTX therapy initiation. The gray band represents the interval to the last follow-up from LD-RTX therapy initiation, and the blue band represents the interval to each relapse from last reinfusion of RTX.

**Supplemental Table 1** Comparison of clinical characteristics of patients with relapses or not during the post-RTX period

| Characteristic | With relapse (*N*=35) | Relapse-free (*N*=73) | *p* value |
| --- | --- | --- | --- |
| Female, *n* (%) | 32 (91.4) | 64 (87.7) | 0.748 |
| Age at onset, y, median (IQR) | 36.0 (27.0–52.0) | 41.0 (28.5–50.5) | 0.657 |
| Serum AQP4-IgG positive, *n* (%) | 33 (94.3) | 59 (80.8) | 0.084 |
| Clinical phenotype of the first attack | | | |
| Myelitis, *n* (%) | 17 (48.6) | 39 (53.4) | 0.684 |
| Optic neuritis, *n* (%) | 14 (40.0) | 20 (27.4) | 0.268 |
| Area postrema syndrome, *n* (%) | 3 (8.6) | 9 (12.3) | 0.748 |
| Acute brainstem syndrome, *n* (%) | 1 (2.9) | 2 (2.7) | 1.000 |
| Cerebral syndrome, *n* (%) | 0 (0) | 2 (2.7) | 1.000 |
| Myelitis and optic neuritis, *n* (%) | 0 (0) | 1 (1.4) | 1.000 |
| Number of attacks pre-RTX, median (IQR) | 3 (1–4) | 2 (1–3) | 0.340 |
| Number of relapses post-RTX, median (IQR) | 1 (1–1) | 0 (0–0) | NA |
| Disease duration pre-RTX, month, median (IQR) | 27.0 (3.0–82.0) | 12.0 (2.0–38.0) | 0.133 |
| Disease duration post-RTX, month, median (IQR) | 44.0 (25.0–60.0) | 32.0 (21.0–43.5) | 0.012 |
| ARR pre-RTX, median (IQR) | 0.9 (0.7–1.4) | 1.4 (0.9–2.4) | 0.009 |
| ARR post-RTX, median (IQR) | 0.3 (0.2–0.6) | 0 (0–0) | NA |
| EDSS pre-RTX, median (IQR) | 4.0 (3.0–5.5) | 3.0 (2.0–4.0) | 0.026 |
| EDSS post-RTX, median (IQR) | 2.5 (1.5–4.0) | 1.5 (1.0–2.3) | <0.001 |
| Side effect, *n* (%) | 5 (14.3) | 17 (23.3) | 0.319 |

Abbreviations: ARR, annualized relapse rate; AQP4, aquaporin-4; EDSS, expanded disability status scale; IQR, interquartile range; RTX, rituximab.
